# Unveiling Graphitic Carbon Nitride in Hexagonal Boron Nitride Epitaxy on Sapphire

**DOI:** 10.1002/smll.76576

**Published:** 2026-10-31

**Authors:** Jakub Iwański, Wiktoria Zajkowska‐Pietrzak, Aleksandra K. Dąbrowska, Mateusz Tokarczyk, Marta Bilska, Marcin Strawski, Magdalena Skompska, Krzysztof P. Korona, Sławomir Kret, Johannes Binder, Andrzej Wysmołek

**Affiliations:** ^1^ Faculty of Physics University of Warsaw Warsaw Poland; ^2^ Institute of Physics Polish Academy of Sciences Warsaw Poland; ^3^ University of Warsaw Faculty of Chemistry Warsaw Poland

**Keywords:** 2D materials, epitaxy, graphitic carbon nitride (g‐C_3_N_4_), hexagonal boron nitride (hBN), interfacial interactions, metal–organic vapor phase epitaxy (MOVPE)

## Abstract

Epitaxial growth of hexagonal boron nitride (hBN) on sapphire is of fundamental importance for the development of van der Waals heterostructures, yet the mechanism of crystallographic alignment across a weakly bonded interface remains unresolved. Here, we demonstrate that graphitic carbon nitride (g‐C_3_N_4_), unintentionally formed during Metal–Organic Vapor Phase Epitaxy (MOVPE) from triethylboron and ammonia precursors, plays a pivotal role in transferring lattice orientation from the Al_2_O_3_ substrate to the overgrown hBN film. High‐resolution transmission electron microscopy, electron energy loss spectroscopy, X‐ray diffraction, photoluminescence, and X‐ray photoelectron spectroscopy consistently reveal a nanometer‐thick interfacial region composed of AlN:O and g‐C_3_N_4_. Despite its partial amorphization upon post‐growth cooling, the layered structure of g‐C_3_N_4_ provides the necessary structural flexibility to accommodate lattice mismatch and transmit crystallographic information across the interface. This finding establishes g‐C_3_N_4_ as an effective buffer enabling epitaxial growth of two‐dimensional hBN on a three‐dimensional substrate. These insights suggest that the presence of carbon at the growth interface, often regarded as detrimental, can in fact be critical for achieving wafer‐scale, high‐quality epitaxial hBN, opening new directions for interface engineering in 2D material synthesis.

## Graphical abstract

Understanding how crystallographic orientation transfers across weakly bonded 2D/3D interfaces is fundamental for van der Waals materials epitaxy. This study reveals that an interfacial graphitic carbon nitride (g‐C_3_N_4_) layer plays a pivotal role in aligning hexagonal boron nitride on sapphire. Its flexible, layered structure effectively transmits lattice information, enabling high‐quality, wafer‐scale 2D heterostructure synthesis.

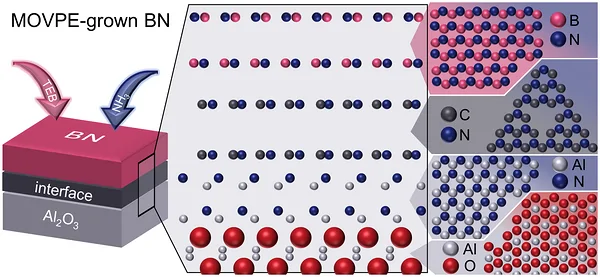

## 1 Introduction

Epitaxial growth techniques enable the fabrication of large‐scale semiconductor materials while preserving the crystallographic orientation of the underlying substrate. This structural coherence extends over macroscopic distances, enhancing the crystalline quality, optical response, and electronic properties of the deposited layers, which is important for industrial applications [1, 2, 3, 4, 5, 6]. In achieving epitaxial growth, the atomic interaction at the interface between the substrate and the growing material plays a crucial role. However, in the case of two‐dimensional materials, which exhibit weak out‐of‐plane van der Waals interactions between atomic layers, the significance of this interaction is less clear. This issue has been reexamined in the framework of remote epitaxy, where a monolayer or few‐layer van der Waals material, such as graphene, is inserted between the substrate and the overgrown film [7, 8, 9, 10, 11]. Remarkably, even in the absence of direct chemical bonding, the epilayer can replicate the substrate's lattice orientation, with the interfacial 2D layer acting as a semi‐transparent barrier with no effect on crystal orientation. This observation implies that long‐range electrostatic fields from the substrate may still govern crystallographic alignment across a van der Waals interface. It also raises fundamental questions about whether the growth of layered materials, such as sp^2^‐bonded boron nitride, can be classified as truly epitaxial and to what extent such materials inherit the substrate's lattice orientation uniformly across its surface, especially when deposited on non‐catalytic substrates. Given the pivotal role of hexagonal boron nitride (hBN) in various van der Waals heterostructures [12, 13, 14, 15, 16, 17], we chose it as a model system to investigate epitaxial growth processes in two‐dimensional materials. Although numerous studies have addressed the growth of hBN on Al_2_O_3_ substrates, the fundamental nature of its crystallographic alignment with the underlying surface remains insufficiently understood [18, 19, 20, 21, 22, 23, 24, 25, 26].

Hexagonal boron nitride is one of the most widely studied two‐dimensional materials, valued for its atomically flat surface, which arises from its graphene‐like crystal structure [27, 28, 29]. In addition to this structural characteristic, its wide bandgap (∼6 eV) makes hBN an excellent insulator [30, 31]. These properties have established hBN as a key material for use as a substrate in the growth of other 2D materials, as well as an encapsulating layer that enhances their optical and electronic performance while improving stability [12, 13, 14]. Consequently, achieving precise control over the epitaxial growth of hBN is not only critical for its own advancement but also for the broader development of van der Waals heterostructures, where it serves as a fundamental building block.

Although adjacent layers in hBN interact primarily through weak van der Waals forces, preferential in‐plane orientations emerge during growth on crystalline sapphire via Metal–Organic Vapor Phase Epitaxy (MOVPE), indicating epitaxial alignment. These orientations are evidenced, for example, by the formation of characteristic surface wrinkles, as shown in Figure 1. The wrinkle pattern arises during post‐growth cooling as a result of thermal expansion coefficient mismatch between hBN and the Al_2_O_3_ substrate [32, 33]. This folding mechanism helps relieve thermal‐induced strain within the layer [34, 35]. As indicated by the red lines, the wrinkles align over extended distances, also across the entire 2‐inch wafer. As demonstrated in Refs. [36, 37], they form along the crystallographic armchair direction (*m*‐plane), suggesting uniform crystallographic orientation across the entire layer. The 120° intersections between the wrinkles are consistent with the expected hexagonal crystal symmetry. Based on the wrinkle pattern, the crystallographic orientation of hBN can be determined, revealing that *m*‐plane in hBN is rotated by 90° in respect to the Al_2_O_3_ substrate.

**FIGURE 1:**
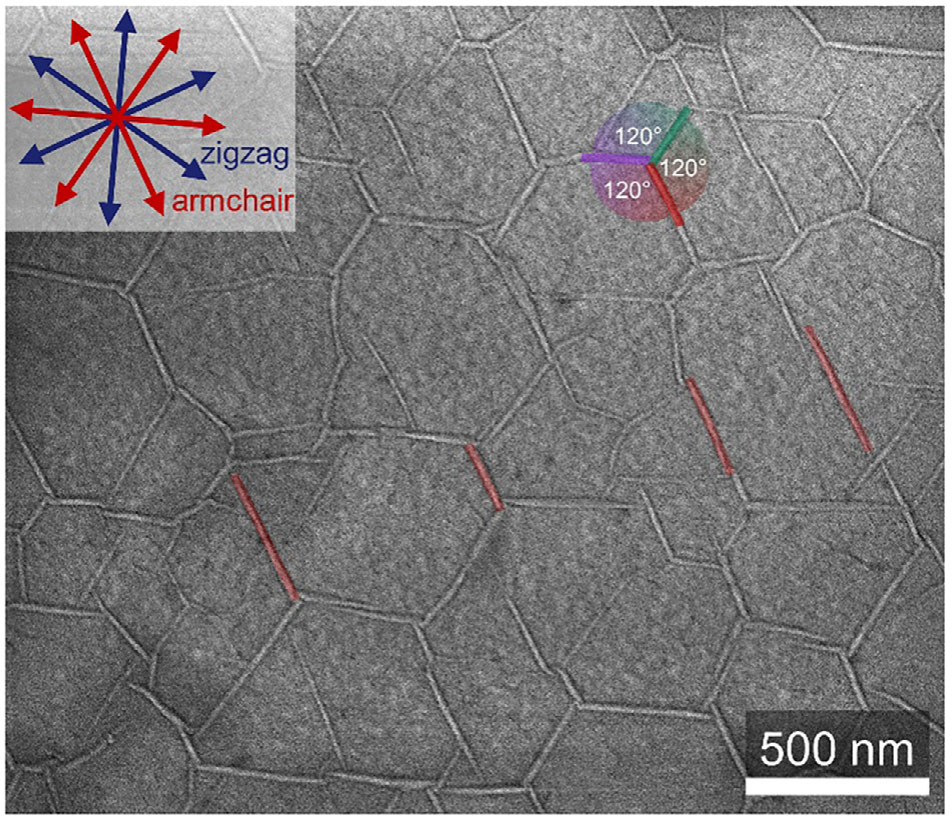
Scanning electron microscope top‐view image of a hexagonal boron nitride (hBN) layer grown on an Al_2_O_3_ substrate. Red lines, which run parallel to each other, highlight the alignment of wrinkles characteristic of an epitaxial layer. Green and purple lines, rotated by ±120°, indicate the orientation of the other wrinkles. The observed 120° intersections between the wrinkles confirm the hexagonal symmetry of the pattern. The inset illustrates the crystallographic orientation of the hBN layer.

Considering the alignment of wrinkles as an indicator for the crystallographic orientation of the hBN layer, and given their long‐range uniformity, it can be inferred that the underlying crystalline substrate imposes a preferred growth direction. In this context, the process may indeed be classified as truly epitaxial. Understanding the mechanism responsible for this substrate‐induced alignment is therefore of fundamental importance. In this study, we demonstrate that graphitic carbon nitride (g‐C_3_N_4_), a layered material with a fractal crystal structure, plays a key role in the orientation of hBN grown by MOVPE on sapphire. The unique structural characteristics of g‐C_3_N_4_, which accommodate variations in interatomic distances, relax lattice mismatch constraints in heteroepitaxy. This suggests that g‐C_3_N_4_ serves as a versatile buffer layer, potentially facilitating the epitaxial growth of two‐dimensional boron nitride.

## 2 Results and Discussion

To investigate the mechanism governing the epitaxial relationship between hBN and Al_2_O_3_, it is crucial to examine the interface between these two materials. Figure 2 presents a cross‐sectional high‐resolution transmission electron microscopy (HRTEM) image of the interface between the epitaxial hBN layer and the sapphire substrate. As shown in Figure 2a, the epitaxial relationship is defined by Al_2_O_3_ [0001] || hBN [0001] and Al_2_O_3_ [112̅0] || hBN [1̅100]. This orientation is consistent with the wrinkle alignment observed in Figure 1 and agrees well with previous reports on epitaxial hBN growth on sapphire using various growth techniques [25, 26]. Notably, a 3–4 nm thick layer is present at the interface between hBN and Al_2_O_3_, appearing partially disordered and lacking crystallinity. This observation is unexpected, given the well‐defined crystallographic alignment of the hBN layer relative to the sapphire substrate. First, hBN atomic layers are bounded by weak, short‐range van der Waals interactions, which suggests that the substrate should have only a limited influence on the crystal orientation. Second, the presence of the interfacial layer increases the separation between hBN and Al_2_O_3_, further reducing the direct interaction. Finally, the amorphous nature of this interfacial region would normally obscure the crystallographic information of the substrate. Taken together, these factors make the observed epitaxial alignment of hBN particularly surprising. It is important to note, however, that the HRTEM analysis is performed at room temperature and does not directly reflect the interface as formed during high‐temperature growth. Upon cooling, strain arising from the thermal expansion mismatch between Al_2_O_3_ and hBN is partially relieved through wrinkle formation. This wrinkling may induce interfacial sliding, potentially leading to bond braking and subsequent amorphization of the mechanically weaker underlying material. No additional electron beam‐induced amorphization was observed under the experimental conditions used in this study. Interestingly, the formation of wrinkles does not fully disrupt the epitaxial alignment of the hBN layer. This is because the interfacial sliding occurs preferentially along specific crystallographic directions, as evidenced by the orientation of the wrinkles along the armchair direction of hBN [36, 37]. Consequently, even though the interface is partially amorphized, the hBN layer maintains a well‐defined alignment relative to the sapphire substrate.

**FIGURE 2:**
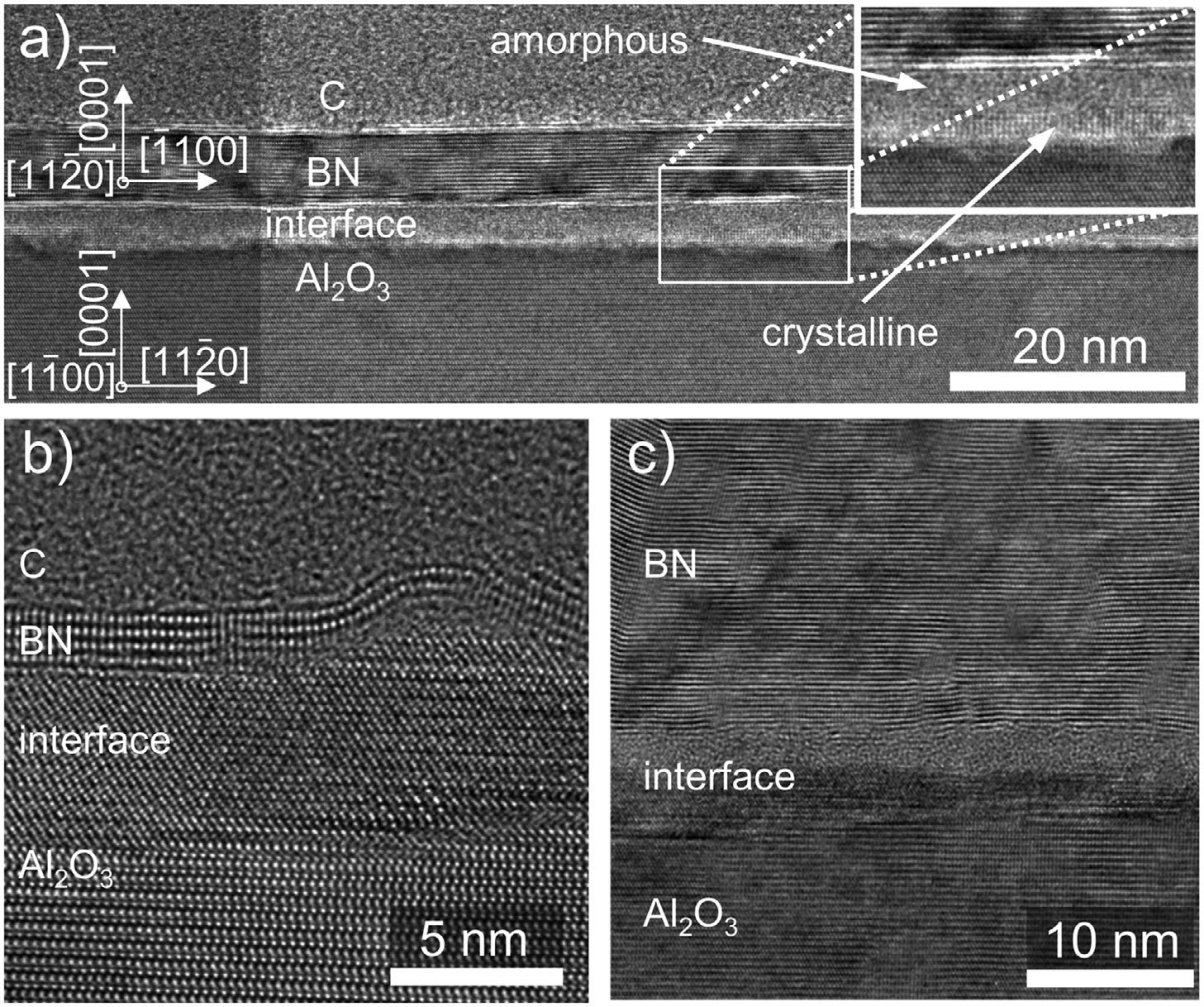
High‐resolution transmission electron microscopy (HRTEM) images of hBN epitaxial layers on an Al_2_O_3_ substrate. (a) Overview of the hBN^8nm^ sample with crystallographic axes of both the layer and the substrate. Inset shows zoom into the interface. The carbon film on top of the hBN layer was deposited in order to enable TEM lamella preparation. (b, c) Magnified views of the interface for hBN^2nm^ and hBN^43nm^ samples.

To study the interface in more detail, we prepared a series of samples grown with varying boron precursor flow rate, which influence both the interfacial structure and the overall growth rate, resulting in different hBN layer thicknesses. The growth time was intentionally kept constant for all samples to ensure comparable thermal exposure of the sapphire substrates during deposition, while variations in the TEB flow were introduced to modify the exposure of the interface to carbon‐containing species originating from the boron precursor. Since TEB constitutes the dominant carbon source in the MOVPE process, changing its flow enabled controlled modifications of the interfacial layer properties, particularly its thickness, as can be seen in Figure 2, without altering other key growth parameters such as temperature or total pressure. Importantly, despite these variations, the formation of the carbon‐rich interfacial layer was consistently observed for all MOVPE‐grown hBN samples on sapphire, indicating that its presence is intrinsically related to the applied growth technology rather than to a specific set of growth conditions. Although the thickness and morphology of this interfacial region vary between samples, it typically extends over a few nanometers and consists of a heterogeneous mixture of amorphous and crystalline phases. Throughout this paper, samples will be denoted by their thickness in superscript notation (e.g., hBN^2nm^ for 2 nm thick hBN layer). For simplicity, the investigated films are referred to as hBN throughout the manuscript, although we note that MOVPE‐grown layered boron nitride may contain mixed sp^2^‐BN polytypes, including both hexagonal and rhombohedral stacking sequences [38, 39]. Since the interfacial layer and epitaxial relationships discussed in this work were found to be independent of the specific BN polytype, a detailed analysis of polytypism is beyond the scope of the present study. As shown in Figure 2b, reducing the growth rate promotes the formation of a well‐defined crystalline interfacial layer. In contrast, increased precursor flow rate results in a thicker amorphous region, as illustrated in Figure 2c, although a crystalline phase at the interface remains discernible. In the following sections, we investigate the structural and chemical characteristics of both the amorphous and crystalline components. As an initial step toward understanding the interfacial composition, we performed elemental analysis. A 52 nm thick hBN film was chosen for this purpose, as it provides sufficient interfacial volume for reliable compositional mapping of both phases.

Figure 3a presents a scanning transmission electron microscopy (STEM) image of the hBN^52nm^ sample. Elemental composition mapping of this region was performed using electron energy loss spectroscopy (EELS), with the results shown in the right panel. Four distinct regions, characterized by different elemental distributions, can be identified. However, the presented results do not permit an accurate determination of the elemental composition of each region. The leftmost region (I) is dominated by aluminum and oxygen, corresponding to the Al_2_O_3_ substrate. The adjacent transition region (II), enriched in aluminum, nitrogen, and oxygen, is attributed to an AlN:O interfacial layer, which has been previously reported in MOVPE‐grown hBN on sapphire [18, 19, 20, 40]. This layer likely forms due to Al_2_O_3_ decomposition in the presence of ammonia at high temperatures [41, 42]. The next region (III), exhibiting an excess of nitrogen and carbon, suggests the presence of a binary C─N compound, as discussed in the next paragraphs, most plausibly graphitic carbon nitride (g‐C_3_N_4_). Finally, the rightmost region (IV) contains only boron and nitrogen, corresponding to the hBN layer. We further analyzed the sample structure using X‐ray diffraction (XRD). In Figure 3b we present 2θ/ω scan performed on the same hBN^52nm^ sample, where several diffraction peaks are identified. The most intense peak at ∼41.7° corresponds to the (0006) reflection of the Al_2_O_3_ substrate for the CuKα radiation [43]. A smaller peak at ∼40° on the shoulder of this strong reflection originates from the same atomic planes but for CuKβ X‐ray lamp line. A broad peak centered at ∼36° is attributed to the (0002) reflection of aluminum nitride [44]. A more detailed scan reveals two components at 2θ_B_ = 35.611° and 2θ_B_ = 36.414° (see Figure S1), deviating from the literature value for pure AlN 36.06° [44]. This suggests that the crystalline interfacial compound should be considered rather as AlN:O, in agreement with EELS compositional analysis. The extracted thickness values for the two types of crystallites with different lattice constants are 2.1 nm and 5.8 nm, respectively. TEM analysis of the hBN^52nm^ sample, which provides local structural information, reveals variations in AlN:O thickness, indicating its inhomogeneity over the interface. In contrast, XRD measurements, which probe a much larger area (mm^2^ scale), capture many crystallites with a wider range of thicknesses. Nevertheless, the overall thickness estimates remain consistent in magnitude for both HRTEM and XRD. The peak at ∼26.7° corresponds to the (0002) reflection of hBN [45]. A more detailed analysis reveals three components of this peak at 26.398°, 26.668°, and 26.731°, corresponding to interplanar distances *d*_0002_ of 3.38, 3.34, and 3.34 Å, respectively. These values are consistent with the expected *c*‐axis lattice parameter of hBN [45]. The lower‐angle peak component is often attributed in the literature to a misoriented or turbostratic phase of layered boron nitride [19, 20]. We did not observe a distinct diffraction peak associated with the carbon‐rich interfacial layer. As discussed previously, this layer most likely undergoes amorphization during the cooling stage following growth, simultaneously with hBN wrinkling, which suppresses its diffraction response. In addition, the expected subtle (002) reflection of g‐C_3_N_4_ originating from areas of better crystallinity observed in some areas in HRTEM images overlaps with the significantly more intense hBN (0002) peak due to their similar *c*‐axis lattice parameters [46, 47].

**FIGURE 3:**
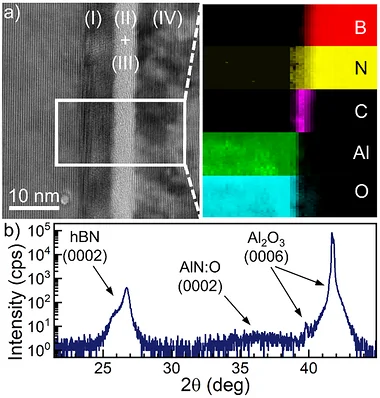
Composition analysis of the hBN^52nm^ sample. (a) Scanning transmission electron microscopy (STEM) image of the interface with marked discussed regions (I)–(IV). The right panel shows the elemental composition of the region outlined by the white rectangle in the STEM image, obtained using electron energy loss spectroscopy (EELS). (b) Wide‐range X‐ray diffraction (XRD) 2θ/ω scan, showing the (0002) peak of hBN at ∼26.7°, the (0002) peak of AlN:O at ∼36.0°, and the (0006) peak of Al_2_O_3_ at ∼41.7°.

The presence of graphitic carbon nitride in MOVPE growth remains largely unexplored. We investigate this possibility, as g‐C_3_N_4_ may play a crucial role in facilitating the transition from conventional sp^3^‐bonded 3D materials to layered sp^2^‐bonded boron nitride in epitaxial growth. To confirm the nature of the carbon‐rich interfacial layer, we conducted a series of experiments.

Figure 4a presents a high‐resolution transmission electron microscopy image of the interface in the hBN^52nm^ sample. In the carbon‐rich region, faint vertical lines can be observed. To analyze these features, we performed a Fast Fourier Transform (FFT) in the area where the lines are present. As shown in Figure 4b, this analysis reveals two distinct peaks, marked by green rings. An inverse FFT (IFFT) was then applied to filter the image, highlighting the structures contributing to these circled peaks (Figure 4c). The vertical stripes become clearly visible, with an interplanar distance of 2.1(1) Å, consistent with the (300) atomic planes of g‐C_3_N_4_, for which *d*_300_ = 2.25 Å [48]. These vertical lines are rarely observed due to the interfacial amorphization induced by wrinkle formation during post‐growth cooling. Nevertheless, similar structures are also observed in other samples. Representative results for the hBN^8nm^ sample are shown in Figure 4d–f. Notably, the image in Figure 4d corresponds to a lamella prepared with a 90° rotation of the structure compared to previous HRTEM images, so Al_2_O_3_ is observed along [112̅0] direction and hBN along [1̅100]. FFT analysis of this sample revealed stripes with a spacing of 1.3(1) Å, corresponding to the (36̅0) atomic planes of g‐C_3_N_4_, where *d*_36̅0_ = 1.30 Å [48]. In the observed two projections rotated by 90° to each other, the following parallel planes can be identified at the interfaces: Al_2_O_3_ (112̅0) || g‐C3N4 (300) || hBN (1̅100) (Figure 4a) and Al_2_O_3_ (1̅100) || g‐C3N4 (36̅0) || hBN (1̅1̅20) (Figure 4d). In both orientations, the calculated lattice mismatch between hBN and g‐C_3_N_4_ amounts to −3.68%, indicating tensile strain in hBN relative to the underlying g‐C_3_N_4_ layer. At the same time, the interface remains weakly bonded along its normal direction through van der Waals interactions. The measured interplanar spacings for hBN, *d*_1̅100_ = 2.2(1) Å and *d*_1̅1̅20_ = 1.3(1) Å, align well with literature values [27, 45]. Figure 5 presents a schematic summary of the crystal structures of g‐C_3_N_4_ and hBN, along with their crystallographic alignment based on experimental results. One can see very good alignment of both crystal structures. The calculated lattice mismatch between hBN and g‐C_3_N_4_ is less than 5%, which is within our experimental uncertainty. This suggests that the carbon‐rich interfacial layer plays a pivotal role in mediating the transition between three‐dimensional and two‐dimensional systems. The characteristic structure of g‐C_3_N_4_, composed of hexagonal rings with periodic voids, likely enables it to flexibly adjust its lattice to the surrounding hBN and AlN:O layers, thereby serving as an effective medium for transmitting crystallographic orientation from the substrate to the overgrown hBN film.

**FIGURE 4:**
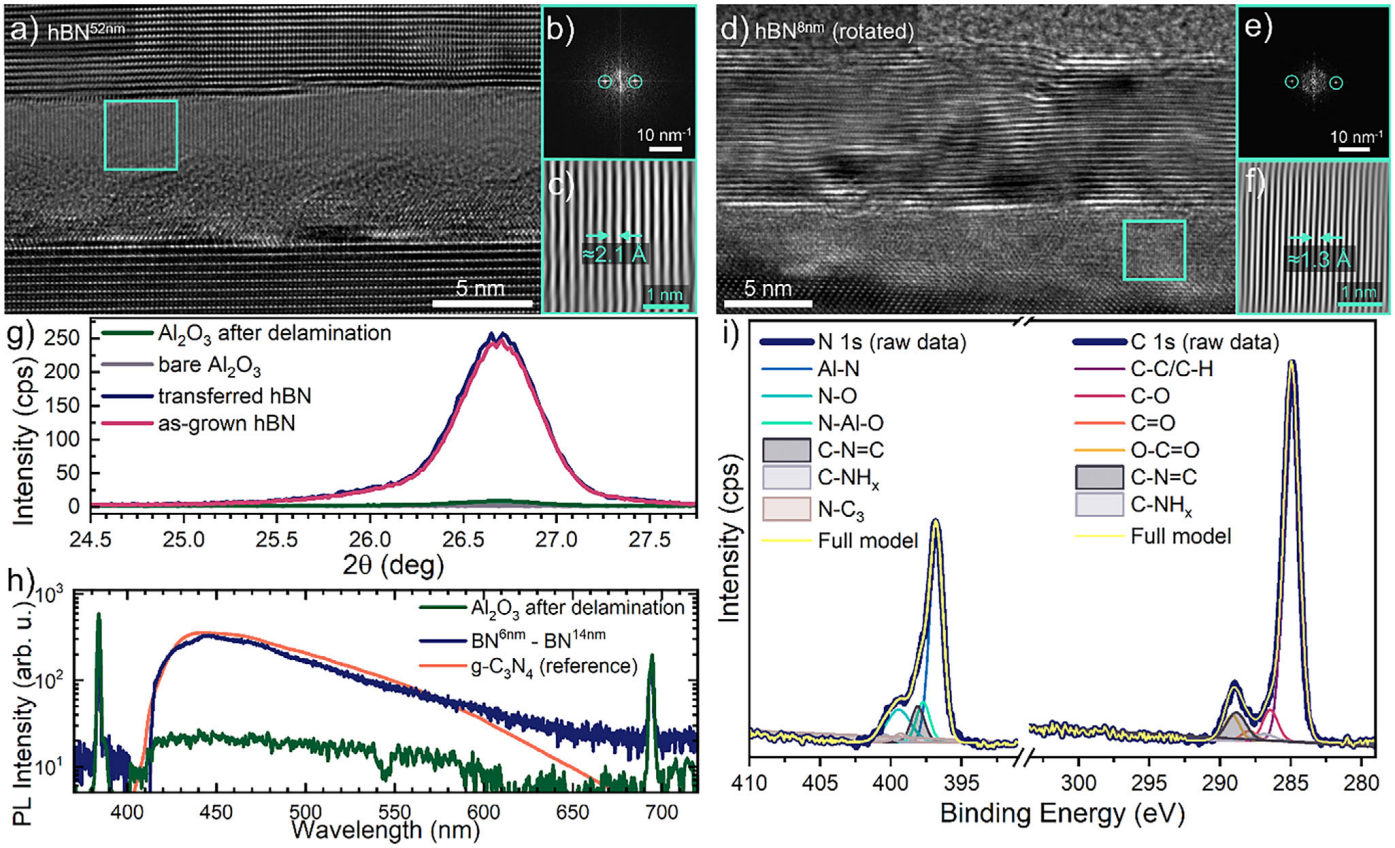
Characterization of the carbon‐rich interfacial layer. (a, d) HRTEM images of the interface. (b, e) Fast Fourier transform (FFT) analysis of the regions outlined by mint frames in (a, d), respectively. (c, f) Filtered signals from the same areas. Panels (a–c) correspond to the hBN^52nm^ sample, while (d–f) show results for hBN^8nm^. (g) 2θ/ω XRD scans for the as‐grown hBN^20nm^ sample (pink), the same sample after delamination and transfer onto a new bare Al_2_O_3_ substrate (navy), the remaining substrate after hBN delamination (green) and bare Al_2_O_3_ as a reference (gray). (h) Photoluminescence spectra of reference g‐C_3_N_4_ powder (salmon), the substrate after hBN^20nm^ delamination (green), and the difference spectrum for hBN^6nm^ and hBN^14nm^ (navy). The peak at 384 nm is related to the residual laser signal, while the peak at 694 nm corresponds to Cr^3+^ transition in the sapphire substrate. (i) High‐resolution XPS spectra of the substrate after hBN^20nm^ delamination, focusing on C‐related (C 1s) and N‐related (N 1s) peaks. Navy lines represent experimental data, while additional lines indicate deconvoluted component peaks, and yellow lines are fitted model.

**FIGURE 5:**
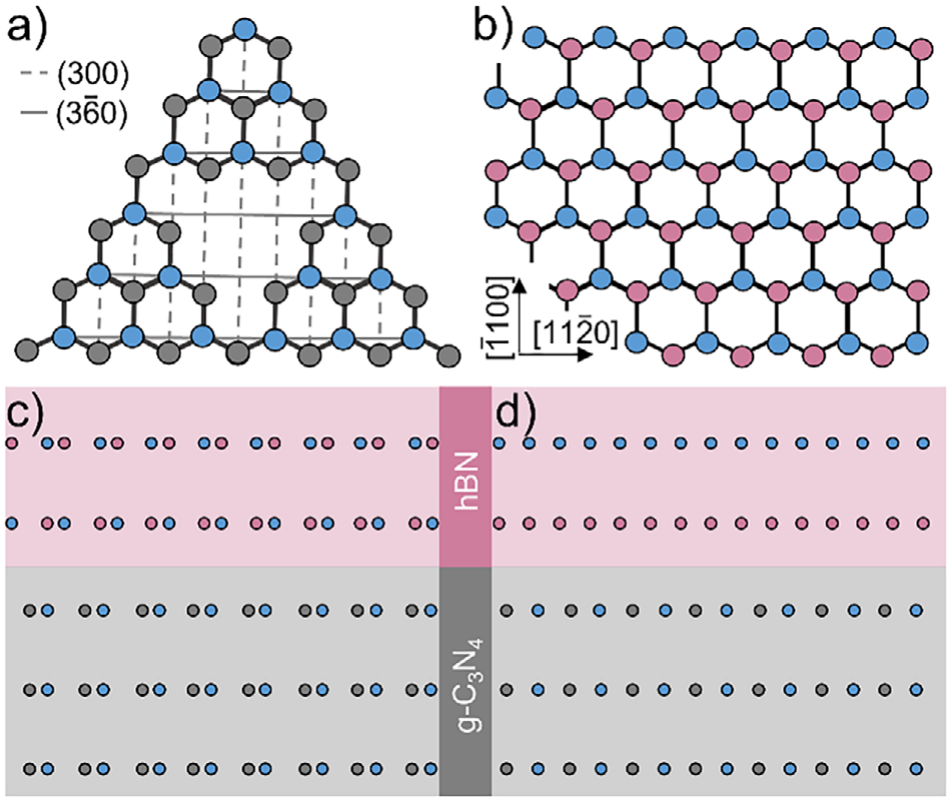
Schematic illustration of extracted crystal alignment. Pink, blue, and gray balls represent boron, nitrogen, and carbon, respectively. (a) g‐C_3_N_4_ crystal monolayer with the lines indicating (300) (dashed line) and (36̅0) (solid line) atomic planes, (b) hBN crystal monolayer, side view of crystal structure along (c) [112̅0] and (d) [1̅100] direction. The crystallographic alignment was derived from experimental data.

Since the lattice parameters of the carbon‐rich interfacial layer observed in HRTEM deviate from the expected values for g‐C_3_N_4_, additional experimental techniques were employed to verify its chemical composition. To this end, the hBN layer was delaminated from the substrate following the procedure described in Ref. [49] and subsequently transferred onto a new Al_2_O_3_ substrate. As shown in Figure 4g, the characteristic ∼27° peak associated with (0002) hBN reflection in 2θ/ω scan is barely discernible on the substrate after delamination. The residual diffraction signal likely stems from minute flakes persisting near the sample edges. It is important to note that the rectangular X‐ray beam used in this measurement spans the entire sample surface, thereby capturing contributions from even trace crystalline remnants, as in this case. The significantly reduced peak intensity‐while maintaining identical position and shape‐suggests a substantial decrease in surface coverage rather than partial separation between atomic layers. On the other hand, the transferred layer retains its original lattice constant and thickness, confirming complete transfer to the new substrate (see Figure S2) and uncovering the interfacial layer on the former substrate. Despite the removal of hBN, as evidenced by XRD, a weak photoluminescence signal is still detectable on the remaining substrate, as illustrated in Figure 4h. This broad emission spectrum closely resembles that of reference, chemically synthesized g‐C_3_N_4_ powder, exhibiting an asymmetric profile with a steep intensity rise around 410 nm, a maximum at ∼440 nm, and a gradual decay extending to 650 nm. Time‐resolved photoluminescence measurements performed on the substrate after delamination revealed an average emission time of (10.8 ± 0.6) ns (see Figure S3), which is in line with reported values for g‐C_3_N_4_ [50, 51]. These spectral similarities further suggest that the interfacial layer consists of g‐C_3_N_4_. During delamination, this layer undergoes degradation, leaving only residual material on the surface (see Figure S4). To further investigate its nature, two samples, hBN^6nm^ and hBN^14nm^, were selected, differing primarily in the thickness of the interfacial layer. Since their hBN defect‐related spectra are similar (see Figure S5), the spectrum of hBN^14nm^ was subtracted from that of hBN^6nm^. This approach was used solely to isolate any additional photoluminescence features that do not originate from the sapphire substrate or from hBN, but instead arise from the differing amounts of interfacial material. Therefore, the analysis is inherently qualitative rather than quantitative. The resulting difference spectrum reveals the photoluminescence signature of the as‐grown interfacial layer, which closely matches that of reference g‐C_3_N_4_ powder, providing further evidence of its chemical composition. To gain additional insight, X‐ray photoelectron spectroscopy (XPS) measurements were performed on a hBN^20nm^ sample where only part of the hBN layer surface had been delaminated. This approach allowed for a direct comparison between the as‐grown hBN layer and the exposed substrate without hBN placed next to it. Such an analysis is particularly important in this study, as carbon is both a key component of the interfacial layer and a common contaminant in XPS measurements. Given that XPS is a surface‐sensitive technique, this direct comparison enabled the differentiation of impurity‐related carbon signals from those originating from the carbon‐rich interfacial material.

As shown in Figure 4i, the carbon‐related XPS spectrum measured on the substrate after hBN delamination consists of multiple peaks corresponding to C─C, C─O, COO, and C═O bonds, which were also detected on the hBN surface (see Figure S6) and are thus attributed to surface contamination. However, additional two components at 287.0 eV and 288.7 eV were necessary to fully deconvolute the signal, providing evidence for C─NH_2_ and C─N═C bonds characteristic of g‐C_3_N_4_ [52, 53]. The g‐C_3_N_4_‐related contribution accounts for approximately 5.5% of the total carbon signal in the detailed spectrum. Given the overall carbon content of 21.9% obtained from the survey spectrum (see Figure S6), the estimated g‐C_3_N_4_ contribution to the total signal is 1.2%. A similar analysis was conducted for the nitrogen‐related N 1s peaks at 398.0, 399.3, and 400.5 eV [53]. The experimental spectra were most accurately reproduced using a model that includes contributions from both AlN:O and g‐C_3_N_4_ (see Figure S6). Importantly, the AlN:O component was independently refined using the AlN:O_ref_ epitaxial sample to ensure that the contribution reflects the actual chemical environment in our material rather than an idealized reference model. The estimated g‐C_3_N_4_ content of the total signal based on the N 1s spectrum is 1.1%, which closely matches the value obtained from the C 1s peak analysis. This consistency suggests that the nitrogen and carbon signals originate from the same compound.

Significant effort was dedicated to demonstrating that the interfacial layer is indeed composed of g‐C_3_N_4_. As discussed above, this layer plays a crucial role in transferring the information about crystallographic orientation from the substrate to the two‐dimensional hBN layer and facilitating the transition between 3D and 2D systems. However, a more detailed explanation is required as to how a material that remains elusive to direct observation by multiple experimental techniques, including HRTEM, can exert such a pronounced effect. To clarify this phenomenon, it is essential to consider the growth conditions. The process occurs at a high temperature of 1300°C–1400°C, at which hydrogen is capable of etching the Al_2_O_3_ substrate, allowing oxygen to escape from the crystal structure [54]. Under these conditions, and in the presence of ammonia, crystalline AlN:O forms from the Al_2_O_3_ substrate. Simultaneously, the interface is also exposed to triethylboron (TEB), a metalorganic boron precursor that contains six carbon atoms per one boron atom. At these temperatures in the presence of hydrogen, TEB undergoes thermal decomposition through a sequence of β‐hydride elimination reactions, yielding boron‐containing intermediates together with hydrocarbon species [55]. Although the exact deposition chemistry is complex and involves multiple reaction pathways, the resulting products remain highly reactive at temperatures exceeding 1000°C and constitute an effective source of both boron‐ and carbon‐containing species [55, 56]. Consequently, the substrate interface is exposed to a chemical environment enriched in carbon, in addition to boron and nitrogen supplied by the precursors. Under these conditions, carbon is highly mobile: it diffuses readily across the hBN surface and, to a lesser extent, between adjacent atomic layers [57]. Because incorporation into the sp^2^‐BN lattice is energetically unfavorable [58, 59], carbon migrates until it either desorbs from the hBN surface or accumulates at the defect‐rich interface formed by substrate etching and AlN:O formation. At these interfacial sites, carbon combines with nitrogen to form g‐C_3_N_4_. The crystal structure of g‐C_3_N_4_, characterized by intrinsic pores in its monolayer (see Figure 5a), enables diffusion of small ammonia molecules toward the AlN:O region, such that AlN:O and g‐C_3_N_4_ develop concurrently. As the g‐C_3_N_4_ layer thickens, it progressively passivates the AlN:O, while the overlying hBN film in turn passivates the g‐C_3_N_4_. With further hBN growth and grain coalescence, the access of carbon and nitrogen to the interface becomes increasingly restricted. Consequently, once the hBN layer reaches sufficient continuity and thickness, the formation of interfacial phases ceases, which is consistent with the observation that the transition layer invariably stabilizes at a thickness of 2–5 nm across different growth conditions.

Following high‐temperature growth, the system is cooled to room temperature. Due to the mismatch in thermal expansion coefficients between the Al_2_O_3_ substrate and the hBN film, the epitaxial layer undergoes strain upon cooling, leading to the formation of wrinkles, as previously reported in the literature [34, 35, 60]. This strain does not only affects the hBN layer but also impacts the g‐C_3_N_4_ interfacial layer. The mechanical stress, combined with the rigidity of hBN, induces bond breaking and structural deformation within the g‐C_3_N_4_ layer, leading to a deterioration of its crystallinity and amorphization. Consequently, in HRTEM images, the observed interface does not reflect its original structure during growth but rather a configuration altered by post‐growth cooling. This structural transformation explains why direct observation of the interfacial layer, crucial for understanding MOVPE growth of 2D materials on conventional substrates, remained challenging for many years.

g‐C_3_N_4_ is a widely studied material in the context of sustainable energy applications [61, 62]. It is typically synthesized from melamine using various techniques, including thermal‐induced self‐condensation, high‐temperature calcination, and chemical vapor deposition, with processing temperatures generally in the range of 500°C–600°C [63, 64, 65]. An important question that remains to be addressed is whether g‐C_3_N_4_ can also be synthesized in our MOVPE system under the applied growth conditions, especially significantly higher growth temperature. To assess the information about thermal stability of g‐C_3_N_4_, thermogravimetric analysis (TGA) is commonly employed, indicating that the material remains stable up to approximately 600°C–700°C [62, 64, 65]. However, this threshold can be further extended through specific strategies, such as boron doping, which strengthens B─N bonds, thereby enhancing both stability and thermal conductivity of the material [66]. Furthermore, given that g‐C_3_N_4_ consists of tri‐s‐triazine units, its thermal decomposition releases species such as HCN, NH_3_, and C_2_N_2_ [67, 68]. The ammonia‐rich environment within the reactor chamber could further promote stability at elevated temperatures. In addition, the overlying hBN film passivates the g‐C_3_N_4_ layer, providing an additional stabilizing effect against thermal degradation. MOVPE is not a conventional technique for g‐C_3_N_4_ synthesis, nor are TEB and NH_3_ typically used as its precursors. Furthermore, no experimental studies have systematically investigated the stability of g‐C_3_N_4_ in an ammonia‐rich atmosphere at temperatures exceeding 1000°C. However, as discussed above, several observations suggest that g‐C_3_N_4_ may not fully decompose under growth conditions used in our study. This hypothesis is further supported by our results, which confirm the presence of this compound at the interface.

A widely adopted strategy in MOVPE growth aimed at enhancing the crystalline quality of hBN involves eliminating carbon‐containing precursors [69, 70, 71]. This approach is motivated by the need to minimize carbon incorporation, which is often considered a structural defect. However, our study highlights the critical role of g‐C_3_N_4_ in the epitaxial growth of hBN. Consequently, we argue that the use of carbon‐containing TEB should not be dismissed right away, as it may facilitate the epitaxial relationship between the substrate and the deposited layer, which is essential for achieving large‐scale, high‐quality thin films. Moreover, the presence of g‐C_3_N_4_ at the interface may have additional functional significance. Due to its favorable photocatalytic properties, g‐C_3_N_4_ is recognized as a potential catalyst for hydrogen generation via water splitting [72]. Hydrogen generation has already been demonstrated for epitaxial MOVPE‐grown hBN layers [73, 74]. In these studies, hydrogen was generated at the substrate‐hBN interface, although the potential role of g‐C_3_N_4_ was not addressed. Our findings provide new insights into this process, suggesting that the epitaxial growth of hBN with an interfacial g‐C_3_N_4_ layer could be exploited for both hydrogen generation and storage.

## 3 Conclusions

In summary, this study investigated the epitaxial growth of hBN on Al_2_O_3_. HRTEM analysis revealed that, although the hBN film does not grow directly on the Al_2_O_3_ substrate and is separated by a nanometer‐thick interfacial layer, it maintains a long‐range epitaxial relationship with the underlying material. Supported by multiple experimental techniques, including HRTEM with FFT analysis, EELS, XRD, PL, and XPS, we demonstrated that the interfacial region comprises two distinct materials: AlN:O, formed through the reaction of Al_2_O_3_ with NH_3_ at high temperatures, and a layered g‐C_3_N_4_. The discovery of this previously unrecognized carbon‐rich layer constitutes the central finding of this work. Its characteristic crystal structure plays a crucial role in mediating the transfer of crystallographic orientation from the sp^3^‐bonded substrate to the sp^2^‐bonded layered material. By revealing the structural and functional role of g‐C_3_N_4_, this study provides new insights into epitaxial mechanisms bridging three‐dimensional and two‐dimensional systems.

## 4 Experimental Methods

### 4.1 Samples

The boron nitride samples analyzed in this study were grown in an Aixtron MOVPE CCS 3×2″ reactor designed for nitride semiconductor growth. Films of various thicknesses were deposited on 2‐inch c‐plane sapphire substrates following a two‐step epitaxy protocol [19, 20]. Triethylboron and ammonia served as the boron and nitrogen precursors, respectively. Each growth sequence began with a 10‐min continuous‐flow buffer layer deposited at 1300°C, followed by flow‐rate modulation epitaxy (FME) performed at 1400°C. The growth parameters were identical for all samples except for the TEB flow rate (changed in the range 5–12 µmol/min), which governed the growth rate as well as the boron and carbon concentrations in the reactor chamber. It also influenced the BN polytypic composition of the final samples.

The AlN:O_ref_ sample was grown in the same reactor using trimethylaluminum and ammonia as the aluminum and nitrogen sources. Its growth involved three stages: (i) deposition of a low‐temperature AlN buffer layer at 890°C for 5.5 min, (ii) a 3‐min recrystallization step at 1210°C, and (iii) an 18‐min high‐temperature growth stage at 1210°C.

The reference g‐C_3_N_4_ sample was prepared by thermal condensation of urea, following the established procedure described in Ref. [50].

### 4.2 Transmission Electron Microscopy

The studies were performed using a FEI Titan Cubed 80–300 transmission electron microscope (TEM) operating at 300 kV and equipped with an image corrector. Electron Energy Loss Spectroscopy (EELS) spectra and Dark Field Image were acquired with GIF camera Gatan Tridiem 866 ERS. Helios Nanolab 600 microscope with a focused ion beam (FIB) system was used for specimen preparation.

### 4.3 X‐Ray Diffraction

Measurements were performed on a Panalytical X'Pert diffractometer equipped with a CuKα radiation source and operated in a parallel‐beam geometry optimized for thin‐film analysis using a parabolic Göbel mirror. To assess the crystalline orientation and quality of the layers, both ω (rocking‐curve) scans and standard 2θ/ω scans were recorded. The combination of these configurations enabled the separation and identification of diffraction peaks originating from the sapphire substrate, the AlN interfacial layer, and the grown hBN. Analysis of the peak positions and their angular offsets allowed determination of the relative crystallographic alignment of the layers, while the full width at half maximum (FWHM) of the diffraction peaks was used to estimate the film thickness.

### 4.4 Photoluminescence

The photoluminescence (PL) was excited using a second harmonic of light from Ti:sapphire (Chameleon Ultra) at the wavelength of 384 nm. The spectral distribution of the PL was analyzed by an ACTON 300 mm spectrometer.

### 4.5 X‐Ray Photoelectron Spectroscopy

X‐ray photoelectron spectroscopy (XPS) measurements were performed on a Kratos Axis Supra spectrometer (Kratos Analytical Ltd., UK), equipped with a monochromatic Al Kα radiation source (1486.7 eV). All data were collected with an analyzer pass energy of 80 eV for survey scans and 20 eV for region spectra, respectively. The instrument work function was calibrated to give a BE of 84.0 ± 0.1 eV for the 4f_7⁄2_ line of metallic gold, and the spectrometer dispersion was adjusted to give a BE of 932.6 eV for the Cu 2p_3⁄2_ line of metallic copper. The effect of sample charging was reduced by co‐axial neutralization system. The occurring shift of energy scale was corrected by setting the main component of C 1s at the literature value of 284.8 eV for adventitious carbon [75]. Peak fitting of the data was performed with use of a Shirley background type. A convolution of GL (30) line shapes was used to fit the individual peaks.

## Author Contributions

**Jakub Iwański**: conceptualization, investigation, writing – original draft, writing – review and editing, funding acquisition, methodology, visualization, formal analysis, project administration, data curation. **Wiktoria Zajkowska‐Pietrzak**: investigation, writing – review and editing, methodology, visualization, formal analysis, data curation. **Aleksandra K. Dąbrowska**: writing – review and editing, investigation, data curation. **Mateusz Tokarczyk**: writing – review and editing, formal analysis, data curation, investigation. **Marta Bilska**: investigation. **Marcin Strawski**: writing – review and editing, methodology, formal analysis, data curation, investigation. **Magdalena Skompska**: resources, writing – review and editing. **Krzysztof P. Korona**: formal analysis, writing – review and editing, investigation, data curation. **Sławomir Kret**: writing – review and editing, supervision, resources. **Johannes Binder**: methodology, funding acquisition, writing – review and editing, resources. **Andrzej Wysmołek**: methodology, supervision, funding acquisition, writing – review and editing, resources.

## Conflicts of Interest

The authors declare no conflicts of interest.

## Supporting information




**Supporting File**: smll76576‐sup‐0001‐SuppMat.pdf.

## Data Availability

The data that support the findings of this study are openly available in RepOD at https://repod.icm.edu.pl/, reference number: https://doi.org/10.18150/IEQQFC
