# Supplementary material for: Unveiling Graphitic Carbon Nitride in Hexagonal Boron Nitride Epitaxy on Sapphire
Source: Small. 2026 Oct 31;22(63):e76576. doi: 10.1002/smll.76576 (PMC13755998; doi:10.1002/smll.76576)
Supplement: Supplementary file 1 [file SMLL-22-e76576-s001.pdf]

Supplementary Information

Unveiling graphitic carbon nitride in hexagonal boron nitride epitaxy on sapphire

Jakub Iwański[1,*], Wiktoria Zajkowska-Pietrzak[2], Aleksandra K. Dąbrowska[1], Mateusz Tokarczyk[1], Marta Bilska[1,2], Marcin Strawski[3], Magdalena Skompska[3], Krzysztof P. Korona[1], Sławomir Kret[2], Johannes Binder[1], Andrzej Wysmołek[1]

[1] *Faculty of Physics, University of Warsaw, Pasteura 5, 02-093 Warsaw, Poland*

[2] *Institute of Physics Polish Academy of Sciences, Aleja Lotników 32/46, 02-668 Warsaw, Poland*

[3] *University of Warsaw, Faculty of Chemistry, Pasteura 1, 02-093 Warsaw, Poland*

*corresponding author: Jakub Iwański (Jakub.Iwanski@fuw.edu.pl)

KEYWORDS: hexagonal boron nitride (hBN), Metal-Organic Vapor Phase Epitaxy (MOVPE), graphitic carbon nitride (g-C_3N_4), interfacial interactions, 2D materials, epitaxy

1. X-ray diffraction of ~36° peak

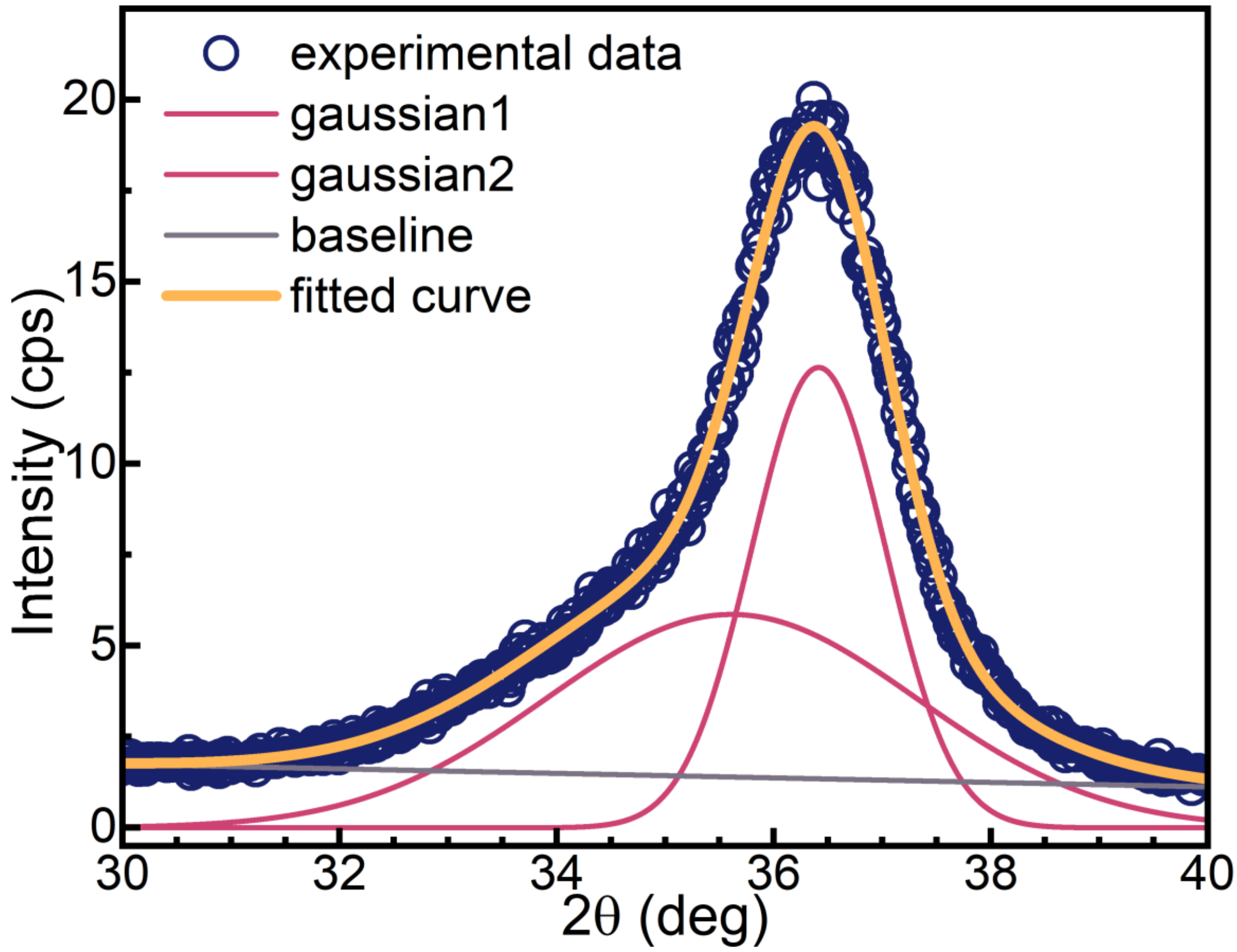

Figure S1: X-ray diffraction (XRD) 2θ/ω scan of the sample shown in Fig. 3 (navy circles) reveals the (0002) reflection of AlN:O at ~36.0°. The signal is more pronounced in this high-resolution measurement, as the offset was adjusted to optimize the diffraction conditions for the AlN:O lattice. The reflection can be deconvoluted into two Gaussian components (pink lines) centered at $2\theta_{B1}$ = 35.611° and $2\theta_{B2}$ = 36.414°, with full widths at half maximum of $FWHM_1$ = 4.02° and $FWHM_2$ = 1.45°, corresponding to lattice constants of c_1 = 5.04 Å and c_2 = 4.93 Å, and associated crystal thicknesses of D_1 = 2.1 nm and D_2 = 5.8 nm, respectively.

2. X-ray diffraction of delaminated layer

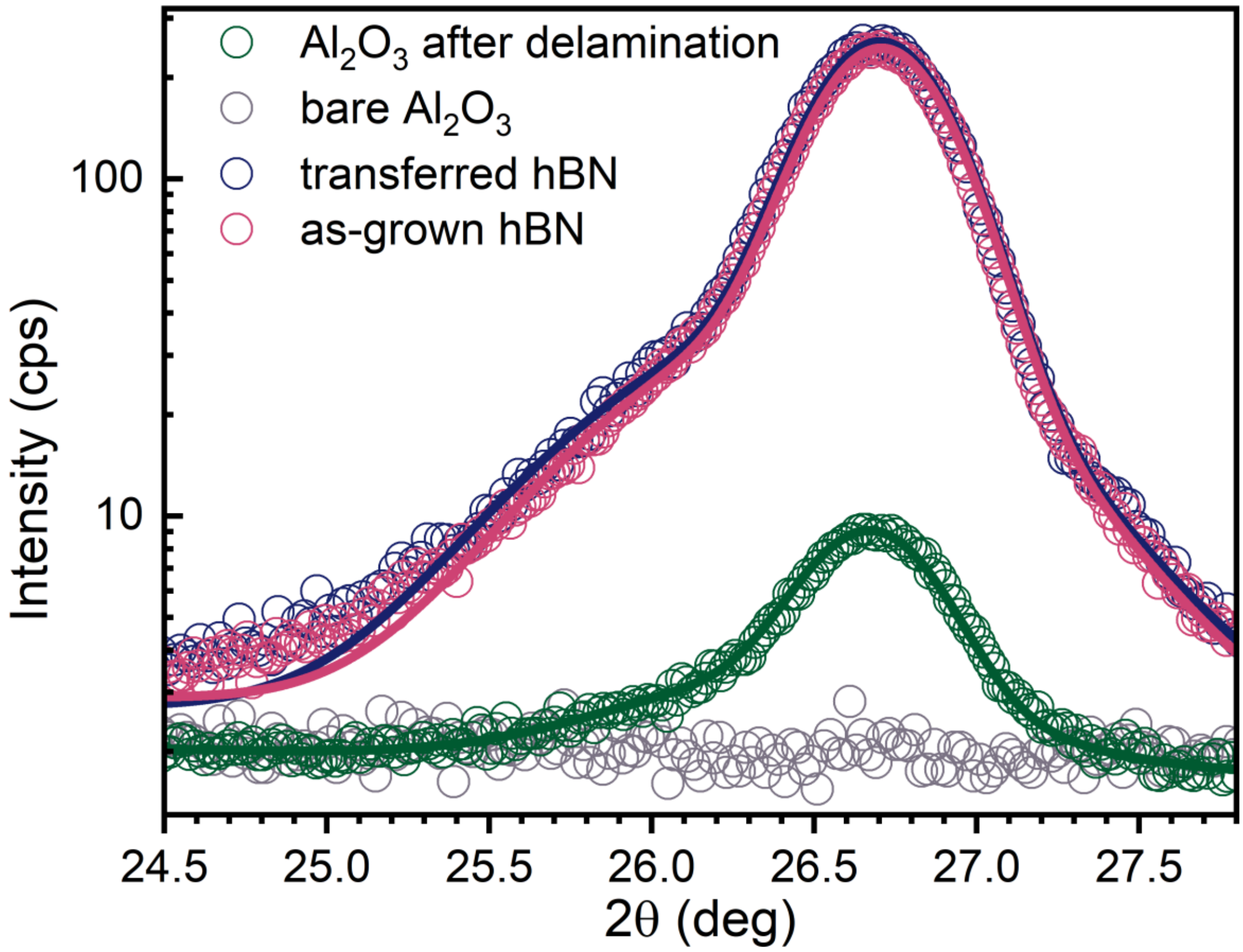

Figure S2: $2\theta/\omega$ XRD scans are shown for the as-grown hBN^{20nm} sample (green), the same sample after delamination and transfer onto a bare Al_2O_3 substrate (blue), the remaining substrate after hBN delamination (red), and bare Al_2O_3 as a reference (gray). Experimental data are plotted as circles, while solid lines of the same color represent Gaussian fits, with the corresponding parameters summarized in Table S1. The reflections associated with Al_2O_3 after delamination, transferred hBN, and as-grown hBN exhibit nearly identical positions and widths, as confirmed by the fitting results. The primary difference arises in peak intensities: the signal from the substrate after delamination is markedly weaker, indicating reduced surface coverage. This residual response likely originates from material remaining near the sample edges, which is still detectable due to the relatively large X-ray beam size (~2 × 10 mm). For better visualization of peak profiles, the data are displayed on a semi-logarithmic scale.

Table S1: Gaussian fitting parameters for the samples presented in Fig. S2. Each diffraction profile was decomposed into two Gaussian components. For every measurement, the upper and lower entries correspond to the first and second component, respectively. The parameter $2\theta_B$ denotes the peak position, FWHM is the full width at half maximum, while d_{0002BN} and D_{BN} represent the calculated interplanar spacing and the thickness of the crystalline fraction.

Sample	$2\theta_B$ (°)	FWHM (°)	d_{0002BN} (Å)	D_{BN} (nm)
as-grown hBN	26.486	1.26	3.37	6.5
	26.711	0.46	3.34	17.8
transferred hBN	26.461	1.32	3.37	6.2
	26.707	0.47	3.34	17.4
Al_2O_3 after delamination	26.441	1.17	3.37	7.0
	26.678	0.46	3.34	17.8

3. Time-Resolved Photoluminescence

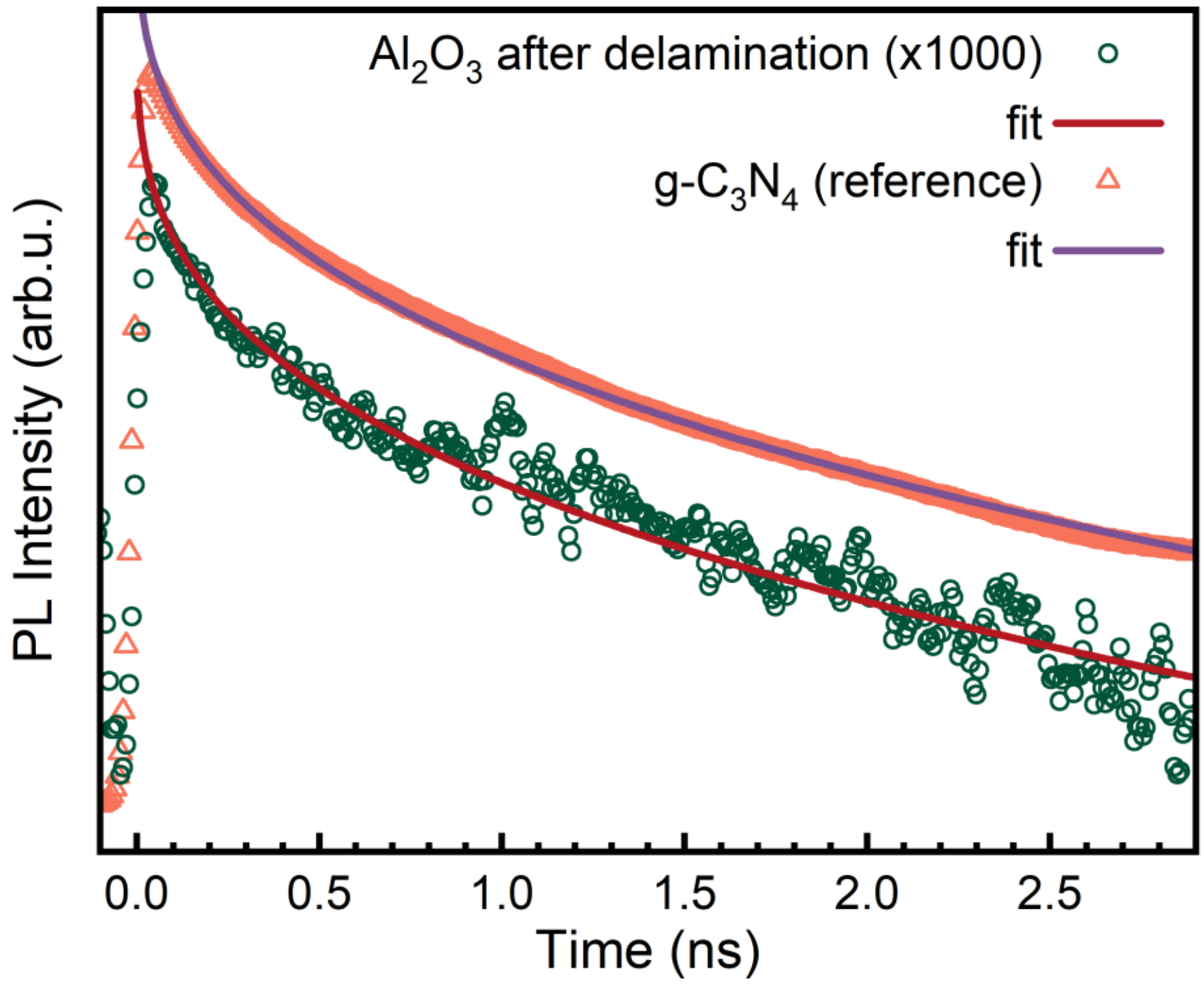

Figure S3: Time-Resolved Photoluminescence traces of substrate after hBN^{20nm} delamination (green circles) and reference g-C_3N_4 (salmon triangles). The vertical axis is presented on a logarithmic scale. The nearly parallel decay profiles observed for both samples suggest that the luminescence signal remaining on the substrate originates from the same carbon-rich phase, providing additional support for the presence of a g-C_3N_4 interfacial layer after delamination. The decay curves were analyzed using the Kohlrausch stretched-exponential model, $\left(I(t) = I(0)e^{-\left(\frac{t}{\theta}\right)^{\beta}}\right)$, which is commonly employed for disordered and heterogeneous systems exhibiting non-single-exponential relaxation dynamics. Such behavior is expected for g-C_3N_4, whose crystal structure and inherently heterogeneous distribution of emissive states give rise to a broad spectrum of relaxation pathways. The partial amorphization of the interfacial layer may further contribute to this behavior. Unlike conventional multi-exponential approaches, the Kohlrausch formalism provides a phenomenological description of the collective relaxation

process and enables estimation of the average emission lifetime. The resulting fits (red and purple lines) yielded (β = 1/3) and (θ = 0.18 ns), corresponding to the Kohlrausch mean relaxation time is $\langle\theta\rangle = \frac{\theta}{\beta}\Gamma(1/\beta) = (1.08 \pm 0.06)\, ns$, and a mean luminescence time $\langle T_{\Phi}\rangle = \int_0^{\infty} t\Phi(t)dt / \int_0^{\infty} \Phi(t)dt = \theta\frac{\Gamma(2/\beta)}{\Gamma(1/\beta)} = (10.8 \pm 0.6)$ ns. Although the acquisition window (3 ns) was shorter than the extracted average lifetime, the two datasets exhibit nearly identical decay characteristics and are well described by the same set of model parameters, further supporting the assignment of the interfacial phase to g-C_3N_4.

4. Transmission Electron Microscopy of delaminated layer

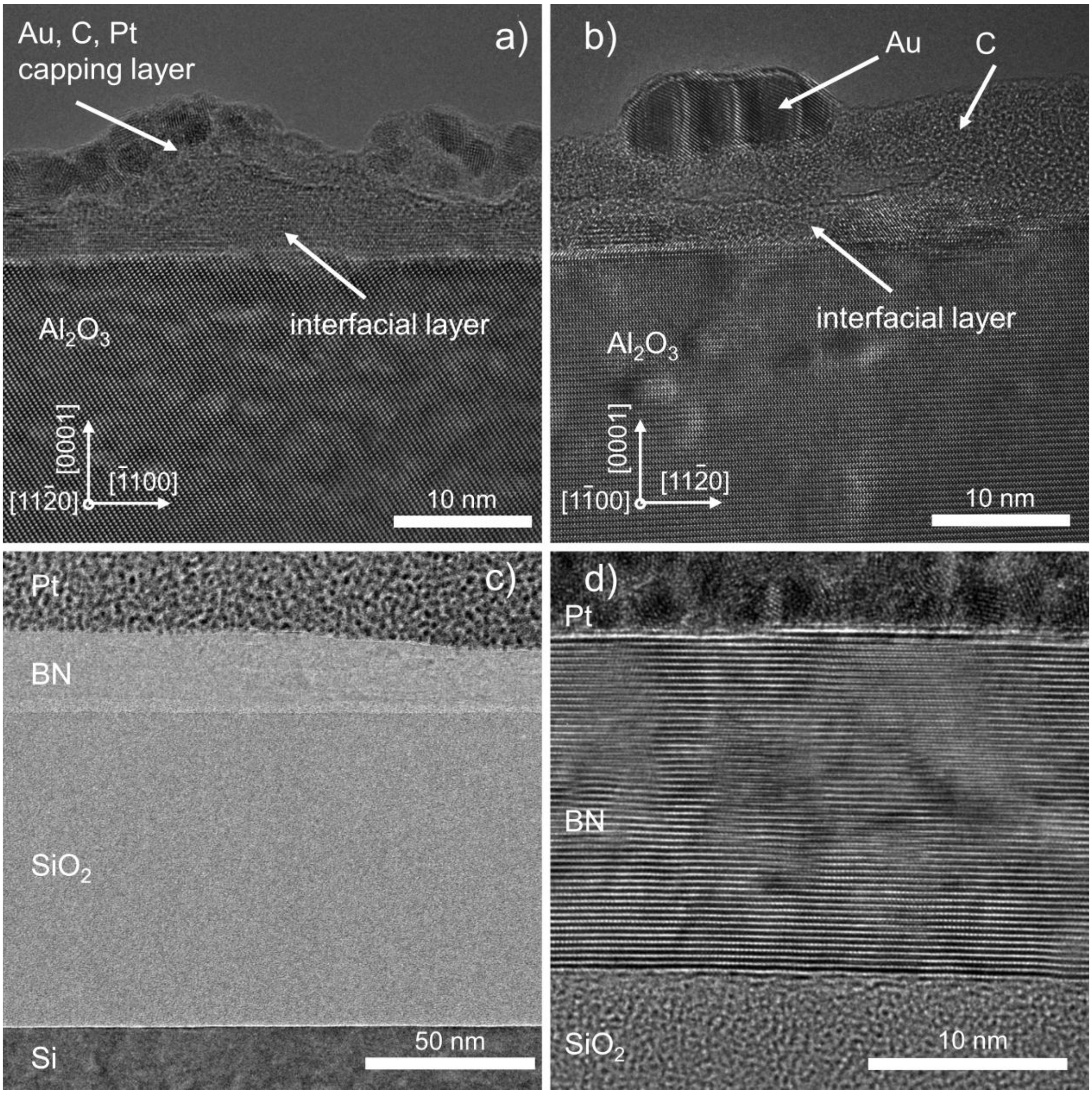

Figure S4: Transmission electron microscopy pictures after layer delamination. Panels a) and b) display the substrate after the delamination process. The lamellae were prepared with a 90° rotation, providing access to different crystallographic orientations. The Al_2O_3 surface still retains the interfacial layer previously identified in TEM images before delamination (see Figs. 2 and 4 in the main text). Above this layer, amorphous carbon, gold, and platinum are

visible, originating from the focused ion beam lamella preparation process. Panel c) shows the hBN layer transferred onto a Si/SiO_2 (90 nm) substrate covered with platinum during lamella preparation. A magnified view of the transferred hBN layer (panel d) reveals that delamination occurred at the g-C_3N_4 - hBN interface, resulting in an atomically smooth SiO_2 - hBN boundary.

5. Photoluminescence spectroscopy of hBN^{6nm} and hBN^{14nm}

The photoluminescence (PL) spectra shown in Fig. S5 were acquired using a 384 nm excitation laser, corresponding to the zero-phonon line (ZPL) of the C380 color center in hBN [1]. A long-pass filter transmitting wavelengths above 400 nm was employed; therefore, the recorded spectra include only the second, third, and fourth phonon replicas of the C380 defect (412, 437, and 468 nm). Clear differences are visible between the responses of the hBN^{6nm} and hBN^{14nm} samples. These variations arise from the distinct hBN film thicknesses and the contribution of the interfacial layer, which can be expressed as:

$$PL(hBN^{6nm}) = \alpha_6 \cdot PL(hBN) + PL(interface) \qquad (S4.1)$$

$$PL(hBN^{14nm}) = \alpha_{14} \cdot PL(hBN) + PL(interface) \qquad (S4.2)$$

where α_6 and α_{14} denote the thickness-dependent scaling coefficients. To isolate the interfacial contribution, the spectra may be subtracted with an appropriate factor $\alpha = \alpha_{14}/\alpha_6$:

$$PL(interface) = \alpha\ PL(hBN^{6nm}) - PL(hBN^{14nm}) \qquad (S4.3)$$

The effect of subtraction is plotted in Fig. S5 (navy). The resulting differential spectrum shows the broad band 420-500 nm, which resembles that of the reference g-C_3N_4 powder (salmon) [2]. The peak at 694 nm is attributed to of Cr^{3+} transition, which is observed in Al_2O_3 or Ga_2O_3 [3].

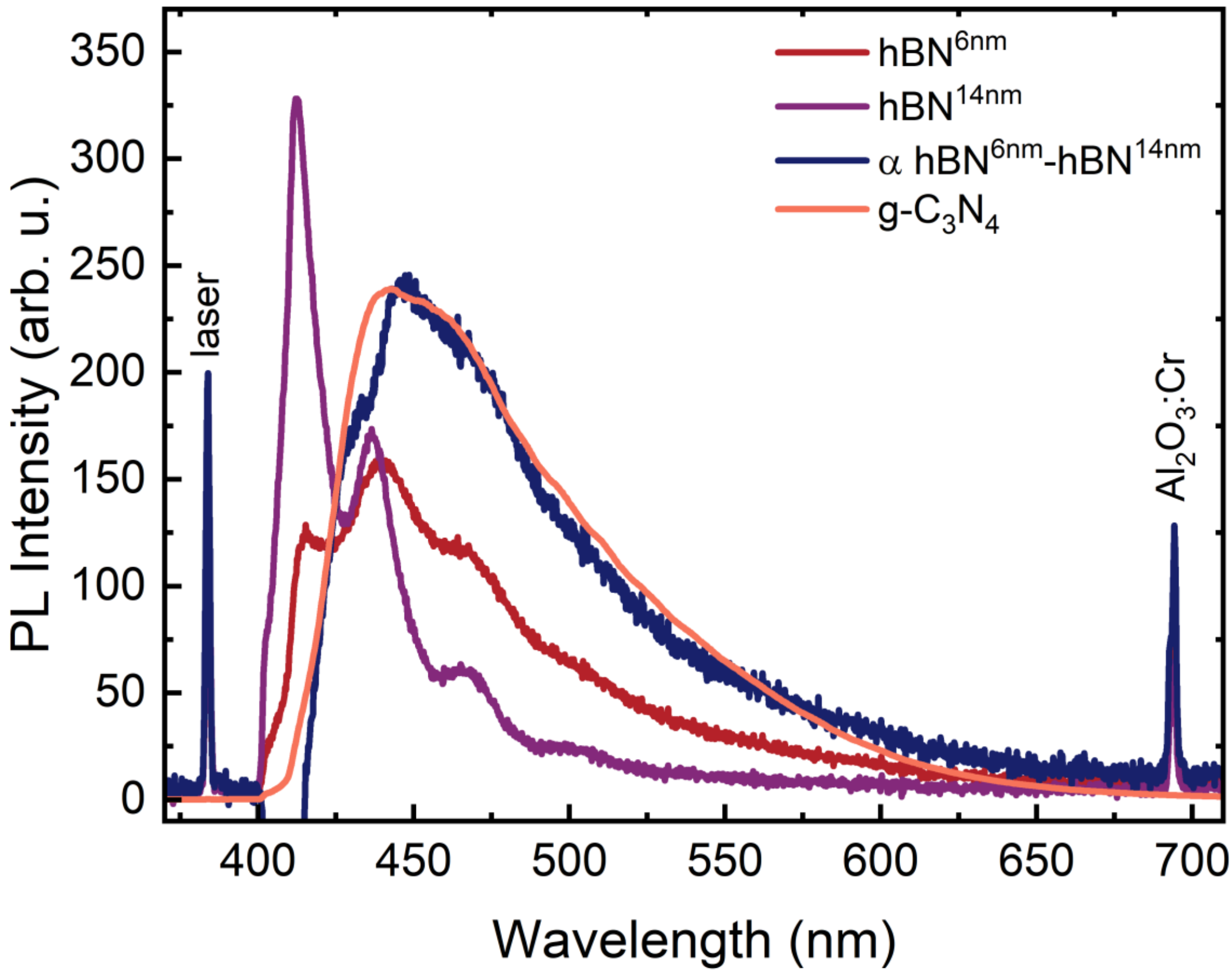

Figure S5: Photoluminescence spectra in the UV range for the investigated samples. The peaks detected in hBN^{6nm} (red) and hBN^{14nm} (purple) are attributed to hBN defect-related emission and the interfacial layer. The differential spectrum (navy) closely resembles that of the reference $g\text{-}C_3N_4$ powder (salmon).

6. X-ray photoelectron spectroscopy detailed analysis

XPS is a recognized surface analysis technique, and this sensitivity is both a disadvantage and an advantage. In the case of materials introduced into the instrument directly from the atmosphere, it is difficult to avoid natural impurities, referred to as adventitious. These are particularly carbon compounds, so the analysis of materials containing this element, the object of our interest, becomes more complicated. That is why we included into the considerations the presence of the adventitious carbon according to the widely discussed by Biesinger group [4–6].

The initial as-grown hBN and AlN:O_{ref} samples exhibit a complex spectrum in the C 1s region (Figs. S6a, c). Adventitious carbon fitted model was applied to these samples and later also transferred to the analysis of the substrate after delamination. In the case of the substrate after delamination, the model did not fully reflect the shape of the recorded spectrum (Fig. S6e). Components were added to the model that can be associated with C–N=C and C–NH bonds, which are reported for g-C_3N_4 and triazine derivatives [7,8]. Including this component allowed us to obtain a representation of the actual result. In parallel, the N 1s region was analyzed. For the reference AlN:O_{ref} sample (Fig. S6d), we obtained a complex spectrum, where, in addition to the dominant Al–N bond, O–Al–N and N–O species appear [9,10]. This model was also transferred to data recorded for the substrate after delamination, and here, too, it did not fully reflect the experimental result (Fig. S5f). The addition and optimization of the components predicted for g-C_3N_4 allowed for the reproduction of the experimental data [7]. The observed N 1s peak for as-grown hBN sample (Fig. S6b) is characteristic for B-N bond observed in hBN [11].

The determined percentage values of the individual components for substrate after delamination sample are presented in Fig. S6g. Summing the components derived from g-C_3N_4

analysis in Fig. S6e-f and including their content in the total content of a given element recorded for the wide scan leads to similar values of 1.2% and 1.1% for C and N, respectively. This convergence of the results confirms our assumption that we are detecting the presence of g-C_3N_4.

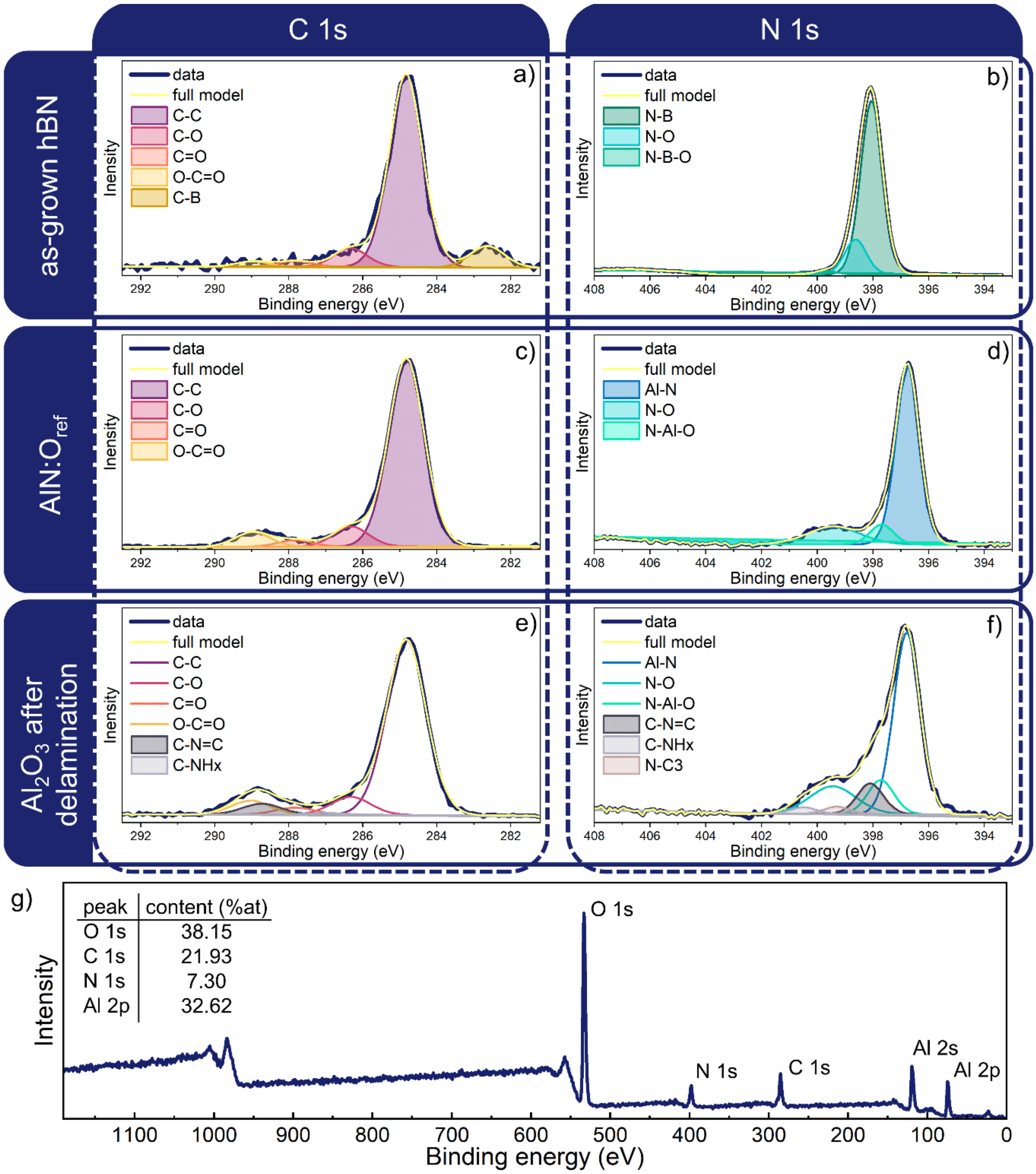

Figure S6: X-ray photoelectron spectroscopy of the sample and reference materials. C 1s spectra for a) as-grown hBN^{20nm}, c) AlN:O$_{ref}$, e) substrate after hBN^{20nm} layer delamination.

The main components of the model are bands originating from aliphatic carbon C–C/C–H and oxidized fragments C–O, C=O, and O–C=O [4]. This system is a typical case of the presence of so-called adventitious carbon, impurities that are difficult to avoid when the sample is exposed to the atmosphere. In the case of the substrate after delamination, C–N=C and C–NHx components were added to the model because the model based on oxidized fragments was not adequate [7,8]. The parallel data collected for the N 1s region for b) as-grown hBN^{20nm}, d) AlN:O_{ref}, f) substrate after hBN^{20nm} layer delamination represent B-N bonds for the hBN standard sample [11] or models containing components assigned to Al–N and Al–O–N bonds for the AlN standard sample [9]. In the as-grown hBN^{20nm} sample, a distinct contribution was also identified in the B 1s region. Importantly, no B 1s signal was detected from the substrate after delamination, confirming that the boron nitride layer does not influence the N 1s spectrum in this state. To correctly interpret the N 1s response of the exposed interface (Fig. S6f), we applied a fitting model containing the characteristic g-C_3N_4 components (C–N=C, N–C3, and C–N–H), which provides an adequate description of the remaining nitrogen-related features. Panel g) presents wide range spectrum, which served us to determine elemental composition of the interface.

REFERENCES

[1] K.P. Korona, J. Binder, A.K. Dąbrowska, J. Iwański, A. Reszka, T. Korona, M. Tokarczyk, R. Stępniewski, A. Wysmołek, Growth temperature induced changes of luminescence in epitaxial BN: from colour centres to donor-acceptor recombination, Nanoscale 15 (2023) 9864–9877. https://doi.org/10.1039/d3nr00578j.

[2] E. Wierzyńska, K. Korytkowska, K. Kazimierczuk, T. Łęcki, K. Zarębska, K.P. Korona, M. Pisarek, B. Furtak, M. Skompska, The Role of Boron Dopant in the Improvement of Electron Transfer in g-C3N4 Photocatalyst, The Journal of Physical Chemistry C 128 (2024) 894–907. https://doi.org/10.1021/ACS.JPCC.3C06373.

[3] K.P. Korona, J. Fink-Finowicki, A. Bohdan, R. Stępniewski, H. Teisseyre, Polarized UV–Visible Photoluminescence from Bulk β-Ga2O3 Crystals, Phys Status Solidi B Basic Res (2025) 2400566. https://doi.org/10.1002/PSSB.202400566;CSUBTYPE:STRING:AHEAD.

[4] M.C. Biesinger, Accessing the robustness of adventitious carbon for charge referencing (correction) purposes in XPS analysis: Insights from a multi-user facility data review, Appl Surf Sci 597 (2022) 153681. https://doi.org/10.1016/J.APSUSC.2022.153681.

[5] D.J. Miller, M.C. Biesinger, N.S. McIntyre, Interactions of CO2 and CO at fractional atmosphere pressures with iron and iron oxide surfaces: One possible mechanism for surface contamination?, Surface and Interface Analysis 33 (2002) 299–305. https://doi.org/10.1002/SIA.1188;REQUESTEDJOURNAL:JOURNAL:10969918;ISSUE:ISSUE:DOI.

[6] J.D. Henderson, B.P. Payne, N.S. McIntyre, M.C. Biesinger, Enhancing Oxygen Spectra Interpretation by Calculating Oxygen Linked to Adventitious Carbon, Surface and Interface Analysis 57 (2025) 214–220. https://doi.org/10.1002/SIA.7376;WEBSITE:WEBSITE:ANALYTICALSCIENCEJOURNALS;REQUESTEDJOURNAL:JOURNAL:10969918;ISSUE:ISSUE:DOI.

[7] D.J. Morgan, Core-level reference spectra for bulk graphitic carbon nitride (g-C3N4), Surface Science Spectra 28 (2021). https://doi.org/10.1116/6.0001083/367021.

[8] V.W.H. Lau, C.F. Lu, N. Putri Wijaya, M. Lutan, Can X-ray Photoelectron Spectroscopy Characterize Photocatalytically Relevant Defects in Graphitic Carbon Nitride? Re-examining Peak Assignment and Quantification Using Model Compounds, Chemistry of Materials 36 (2024) 9762–9774. https://doi.org/10.1021/ACS.CHEMMATER.4C01902.

[9] L. Rosenberger, R. Baird, E. McCullen, G. Auner, G. Shreve, XPS analysis of aluminum nitride films deposited by plasma source molecular beam epitaxy, Surface and Interface Analysis 40 (2008) 1254–1261. https://doi.org/10.1002/SIA.2874;WGROUP:STRING:PUBLICATION.

[10] E. Radhika, P. Dobbidi, XPS and dielectric studies of phase pure AlN-BN composite ceramics using powder bed, Materials Science and Engineering: B 297 (2023) 116783. https://doi.org/10.1016/J.MSEB.2023.116783.

[11] A.A. Tonkikh, E.N. Voloshina, P. Werner, H. Blumtritt, B. Senkovskiy, G. Güntherodt, S.S.P. Parkin, Y.S. Dedkov, Structural and electronic properties of epitaxial multilayer h-BN on Ni(111) for spintronics applications, Scientific Reports 2016 6:1 6 (2016) 23547-. https://doi.org/10.1038/srep23547.
